# Clinical Utility of Cardiovascular Magnetic Resonance in Hypertrophic Cardiomyopathy

**DOI:** 10.1186/1532-429X-14-13

**Published:** 2012-02-01

**Authors:** Martin S Maron

**Affiliations:** 1 Hypertrophic Cardiomyopathy Center, Division of Cardiology, Tufts Medical Center, Boston, MA

**Keywords:** Hypertrophic cardiomyopathy, cardiovascular magnetic resonance, heart failure, sudden death, fibrosis

## Abstract

Hypertrophic cardiomyopathy (HCM) is characterized by substantial genetic and phenotypic heterogeneity, leading to considerable diversity in clinical course including the most common cause of sudden death in young people and a determinant of heart failure symptoms in patients of any age. Traditionally, two-dimensional echocardiography has been the most reliable method for establishing a clinical diagnosis of HCM. However, cardiovascular magnetic resonance (CMR), with its high spatial resolution and tomographic imaging capability, has emerged as a technique particularly well suited to characterize the diverse phenotypic expression of this complex disease. For example, CMR is often superior to echocardiography for HCM diagnosis, by identifying areas of segmental hypertrophy (ie., anterolateral wall or apex) not reliably visualized by echocardiography (or underestimated in terms of extent). High-risk HCM patient subgroups identified with CMR include those with thin-walled scarred LV apical aneurysms (which prior to CMR imaging in HCM remained largely undetected), end-stage systolic dysfunction, and massive LV hypertrophy. CMR observations also suggest that the cardiomyopathic process in HCM is more diffuse than previously regarded, extending beyond the LV myocardium to include thickening of the right ventricular wall as well as substantial morphologic diversity with regard to papillary muscles and mitral valve. These findings have implications for management strategies in patients undergoing invasive septal reduction therapy. Among HCM family members, CMR has identified unique phenotypic markers of affected genetic status in the absence of LV hypertrophy including: myocardial crypts, elongated mitral valve leaflets and late gadolinium enhancement.

The unique capability of contrast-enhanced CMR with late gadolinium enhancement to identify myocardial fibrosis has raised the expectation that this may represent a novel marker, which may enhance risk stratification. At this time, late gadolinium enhancement appears to be an important determinant of adverse LV remodeling associated with systolic dysfunction. However, the predictive significance of LGE for sudden death is incompletely resolved and ultimately future large prospective studies may provide greater insights into this issue. These observations underscore an important role for CMR in the contemporary assessment of patients with HCM, providing important information impacting diagnosis and clinical management strategies.

## Introduction

Hypertrophic cardiomyopathy (HCM) is the most common genetic cardiomyopathy (prevalence of 1:500 in the general population) caused by mutations in genes encoding proteins of the cardiac sarcomere [1-5]. A clinical diagnosis of HCM is confirmed when unexplained increased LV wall thickness is imaged (range 13-60 mm with average 22 mm) in the presence of a nondilated LV chamber [1,3,6]. HCM is a global disease affecting many races and equally by gender [7,8]. Despite a diverse pattern of phenotypic expression and clinical course, HCM is compatible with normal life expectancy in the vast majority of patients [9-11]. However, a small but important subset of HCM patients remain at increased risk of adverse disease complications such as sudden death, progressive heart failure symptoms or stroke [1,12-15].

**Figure 1:**
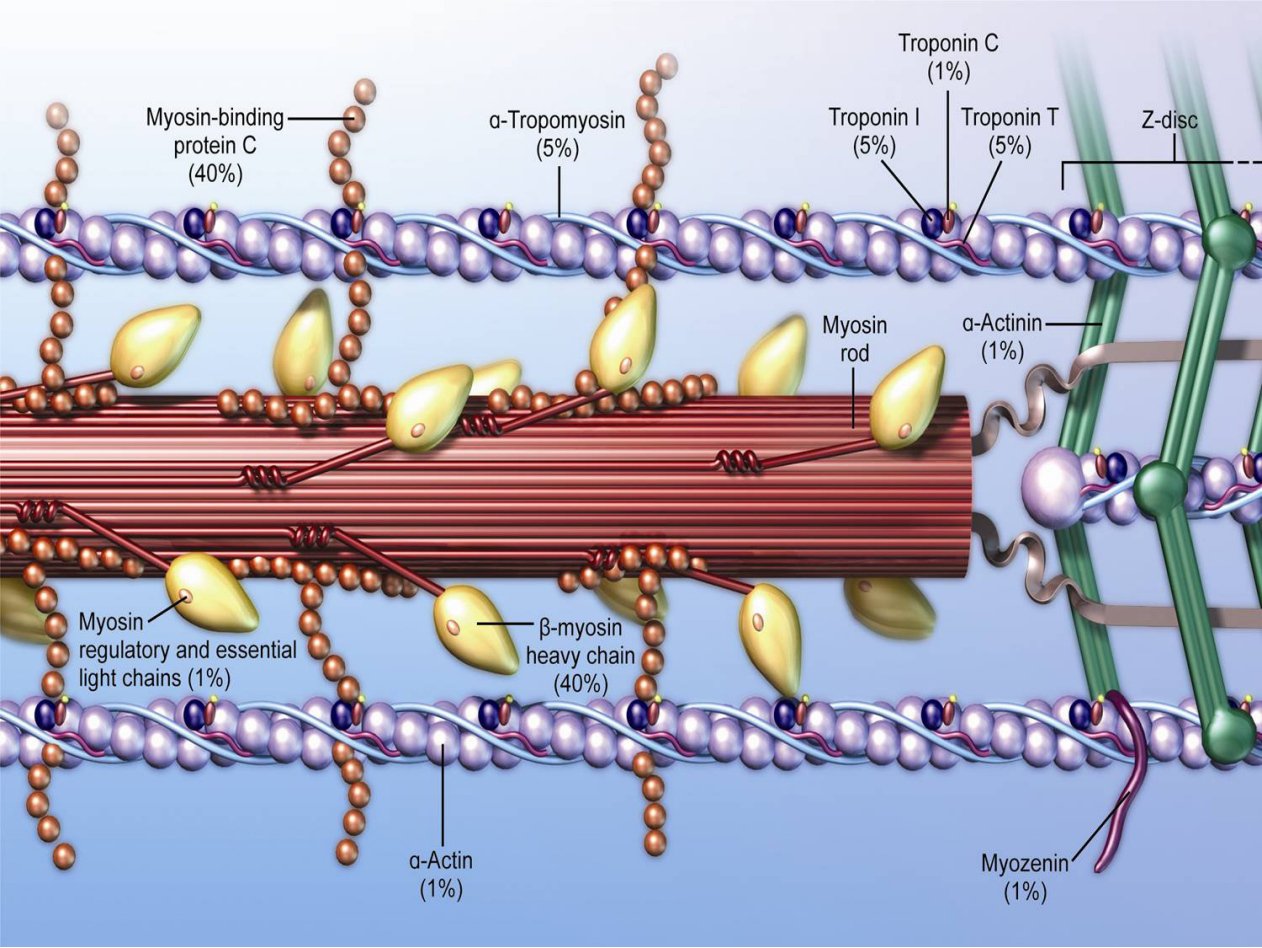
**Cardiac sarcomere showing the location of known disease-causing genes for HCM. Adapted from Wheeler et al.**[20] Not shown are genes previously linked to HCM, but with lesser degrees of evidence for disease causing: titin, vinculin, muscle LIM protein, telethonin, cardiac ankyrin repeat protein, calreticulin 3, calsequestrin 2, phospholamban, ryanodine receptor 2.

*Genetics*. HCM is caused by vast genetic heterogeneity with > 1,400 mutations in 13 or more genes encoding contractile proteins of the cardiac sarcomere (or in sarcomere-associated proteins) with cardiac β-myosin heavy chain (MYH7) and cardiac myosin binding protein C (MYBPC3)(Figure 1) the two most common sarcomere mutant genes, each accounting for the majority of HCM [5,16-18]. Mutations responsible for HCM are transmitted in an autosomal dominant manner in which each offspring of an affecting family member has a 50% chance of inheriting the mutation. Nearly all patients who inherent a disease-causing mutation will demonstrate evidence of the disease with increased wall thickness by early adulthood. However, select mutations can demonstrate substantial variability in age-related penetrance, resulting in delayed expression of the phenotype into the third decade of life, or even beyond to mid-life [19].

Currently, clinic genetic testing can identify a sarcomere protein mutation in 50-60% of patients with a phenotype of HCM [17,20,21]. Therefore, the genetic basis of a substantial number of patients with phenotypic evidence of HCM remains uncertain [20]. However, in those HCM index patients in whom a mutation is identified, genetic testing can then provide the opportunity to identify relatives at-risk of developing disease [17,20-22]. For relatives identified as having a mutation but without left ventricular hypertrophy (ie., genotype positive/phenotype negative) it is likely that this patient will develop phenotypic evidence of disease at some point in there clinical course, prompting recommendations for continued imaging surveillance [23]. For those family members who do not carry the mutation there is no future risk of developing HCM and therefore no further testing is necessary [17,21].

Genetic testing can also differentiate a number of uncommon systemic metabolic diseases, which can manifest a pattern of LV hypertrophy nearly identical to that of sarcomeric HCM but which have different treatment strategies. These diseases are due to mutations in encoding the γ2 regulatory subunit of adenosine monophosphateactivated protein kinase (*PRKAG2)*, lysosome associated membrane protein (*LAMP2) *and the recessive disorder Fabry disease caused by mutations in the gene α-galactosidase (GLA) [17,21,24].

Currently, identifying HCM patients at risk for adverse disease-related events including sudden death cannot be predicted based on specific mutations [16,17,20,21,25]. As a result, management decisions such as ICD therapy for primary prevention cannot be made solely based on results derived from genetic testing [21,26]. Furthermore, the observation that disease expression among first-degree family members with HCM can be dramatically different as well as the fact that specific HCM phenotypes (ie., apical HCM, apical aneurysms, end-stage HCM, etc.) have diverse mutations associated with them, suggest there is no clear relationship between mutation and phenotype [27]. The explanation for these observations may be due to a number of less well-understood factors, such as how modifier genes and environment also contribute to HCM disease expression [4].

### Cardiovascular magnetic resonance (CMR)

**Figure 2:**
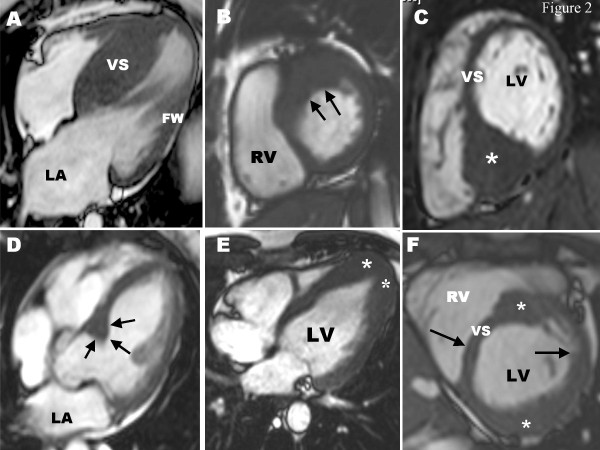
**CMR end-diastolic images demonstrating diverse patterns of LVH in HCM**. **(A) **involving ventricular septum (VS), but sparing the LV free wall (FW); **(B) **hypertrophy of the basal anterior free wall and a portion of the contiguous anterior septum, representing the most common area of LV wall thickening in HCM; **(C) **massive hypertrophy (wall thickness, 33 mm) limited to basal posterior ventricular septum (asterisk); **(D) **focal area sharply confined to basal anterior septum (arrows); **(E) **localized to LV apex (asterisks); **(F) **segmental LV hypertrophy of the basal anterior septum and anterolateral wall (asterisks), separated by regions of normal LV thickness (arrows). Adapted with permission, from Maron MS et al.[29] FW = free wall; LA = left atrium; LV = left ventricle; RA = right atrium; RV = right ventricle; VS = ventricular septum.

Cardiovascular magnetic resonance (CMR) has recently emerged as an important imaging technique by offering a number of unique strengths which make it particularly well suited to provide detailed characterization of the HCM phenotype and therefore an important aid for diagnosis and potentially prognosis [28-34]. CMR can provide 3-dimensional tomographic imaging with high spatial and temporal resolution images of the heart, in any plane and without ionizing radiation (Figure 2) [30]. Contemporary functional cine CMR imaging sequences (ie., steady-state free precession) allow clear delineation of the endocardial and epicardial borders by producing sharp contrast between the interface of darkened myocardium and bright blood pool, which permit for precise wall thickness measurements in any location of the LV myocardium [34]. Furthermore, CMR provides truly tomographic imaging by acquiring a stack of short-axis images (with no interslice gap) with full ventricular coverage and therefore the opportunity to inspect the LV myocardium for limited, focal hypertrophy [29,35]. CMR images are not encumbered by the same limitations inherent in echocardiographic imaging, such as poor image quality related to thoracic or pulmonary parenchyma or inaccurate wall thickness measurements due to short-axis obliquity [30]. Lastly, myocardial fibrosis can be identified with contrast-enhanced CMR sequences after the intravenous injection of gadolinium images, which may select patients at increased risk of adverse disease consequences [36-39].

### CMR for Diagnosis

**Figure 3:**
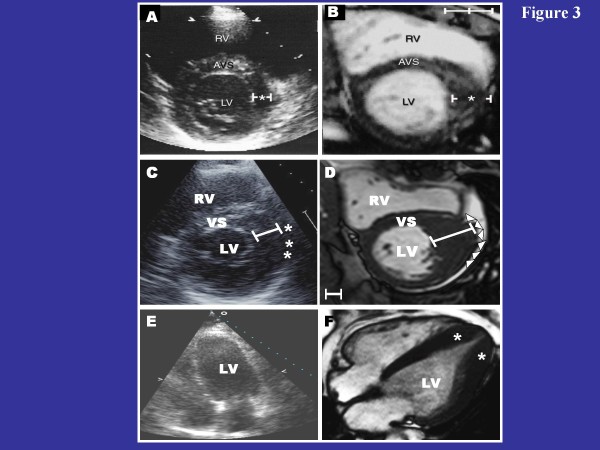
**CMR can identify segmental LV hypertrophy that may not be reliably visualized by two-dimensional echocardiography**. (**A) **normal 2-D echocardiogram in a patient with a family history of HCM; (**B) **this same patient then underwent CMR, which reveals an area of segmental hypertrophy in the anterolateral LV wall (asterisk) consistent with a diagnosis of HCM. Reproduced with permission of American Heart Association; from Rickers C et al.;[35] (**C) **two-dimensional echocardiographic end-diastolic basal short-axis view demonstrates a maximal LV wall thickness of 18 mm in the anterolateral free wall consistent with the diagnosis of HCM; **(D) **in the same patient, an end-diastolic short-axis CMR at the same level of LV shows a focal area of massive LV hypertrophy (35 mm) in the same region of the LV wall reported to be 18 mm by echocardiography. The finding of massive hypertrophy by CMR, characterized this patient as high risk, and prompted recommendation for ICD therapy for primary prevention of sudden death. Reproduced with permission, from Maron MS et al.;[42]**(E) **echocardiography was considered non-diagnostic; **(F) **in the same patient, CMR clearly demonstrates segmental hypertrophy confined to the LV apex, consistent with a diagnosis of apical HCM. Reproduced with permission, from Moon et al.[43] LV = left ventricle; RV = right ventricle; VS = ventricular septum.

Traditionally, two-dimensional echocardiography has been the primary imaging modality used for a clinical diagnosis of HCM by demonstrating an otherwise unexplained increase in LV wall thickness (avg., 21-22 mm) in the presence of a nondilated LV cavity [1,40,41]. However, over the last decade, several important observations have emerged related to the role of CMR in the diagnosis of HCM. First, when echocardiographic images are technically suboptimal and nondiagnostic, CMR has the distinct advantage of defining LV wall thickness measurements with high-resolution imaging [33,41]. Second, CMR has proved advantageous in identifying the presence and/or magnitude of LV hypertrophy, particularly when regions of increased wall thickness are completely (or predominantly) limited to only focal areas of the LV wall such as the anterior free wall, posterior septum and apex [29,35,42-45]. In one recent study, an important subset of patients with HCM were ultimately diagnosed with this disease only after LV hypertrophy was recognized in the anterolateral wall by CMR [35], suggesting this may be the most common area of the LV wall in which hypertrophy may be missed by echocardiography (Figure 3a and 3b). This observation can be explained due to the difficulty in differentiating the lateral epicardial border of the LV myocardium from the adjacent thoracic parenchyma in the short-axis orientation due to loss of spatial resolution in that portion of the imaging sector by echocardiography. For similar reasons, defining the epicardial border of the posterior septum in the area of insertion of the RV free wall can also be difficult with echocardiography [29]. Lastly, in some patients, thoracic and pulmonary parenchyma may limit the ability of echocardiocardiography to accurately define the endo or epicardial border of the apex, while CMR is not limited by such constraints (Figure 3c and 3d) [43-45]. These observations also support the wider use of CMR in screening family members [23].

### Phenotype Characterization

#### Pattern and distribution of LV hypertrophy

The most common location for increased LV wall thickness in HCM patients is the confluence of the basal anterior septum with the contiguous anterior free wall (ie., one o'clock position in the LV short-axis image; Figure 2b) [29]. Hypertrophy involving both of these segments is present in close to 70% of HCM patients and therefore this region of the LV constitutes the most frequent location for increased wall thickness in this disease. Due to the aforementioned limitations in accurately identifying the borders of these wall segments with lower spatial resolution echocardiography, this CMR observation in HCM differs from that of the impression of echocardiography in which the predominant area of hypertrophy was the basal anterior septum (ie., 12 o'clock position) [40]. In a population of HCM patients, the next most common region for increased wall thickness is the posterior septum at the mid-LV level (Figure 2c) [29].

The majority of HCM patients have diffuse hypertrophy involving more than 50% or greater of the LV myocardium (Figure 2a). Notably, a substantial minority of HCM patients have particularly focal or regional areas of increased wall thickness involving only one or two LV segments most commonly involving the basal anterior septum (Figure 2d) but also the anterolateral free wall, posterior septum and apex (Figure 2e) [29]. In addition, LV mass is normal in a substantial portion of HCM patients with limited, focal hypertrophy [46]. These CMR-based observations emphasize an important principle that even very limited hypertrophy (with normal LV mass), can be consistent with a clinical diagnosis of HCM [29,46].

#### Right ventricle

**Figure 4:**
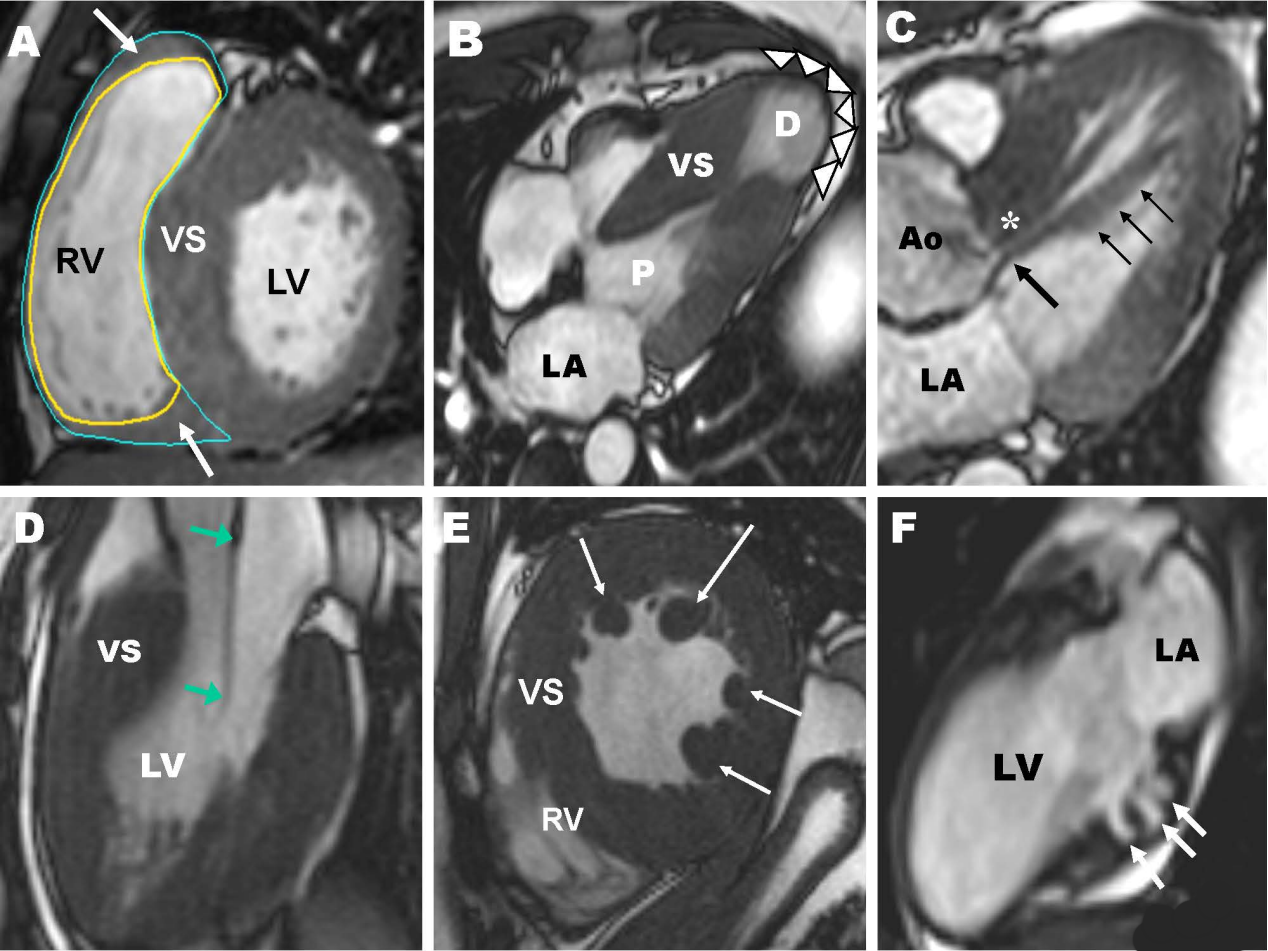
**CMR end-diastolic images demonstrating diversity of the phenotypic expression within HCM**. **(A) **increased wall thickness in the superior segment (thin arrow) and extreme hypertrophy of the inferior segment (thick arrow) of the RV wall; Reproduced with permission, from Maron MS et al.[29]**(B) **medium-sized LV apical aneurysm (arrowheads) and maximal LV wall thickening at mid-ventricular level with muscular apposition of septum and LV free wall producing distinct proximal (P) and distal chambers; Reproduced with permission, from Maron MS et al.[50]**(C) **anomalous insertion of papillary muscle (thin arrows) directly into the anterior leaflet of the mitral valve (thick arrow) (in the absence of chordae tendinae) producing obstruction to blood flow from the apposition of the papillary muscle and basal ventricular septum (asterisk); **(D) **extraordinarily long anterior mitral valve leaflet measuring 33 mm; PML is of normal length (although not well visualized in this frame); Reproduced with permission, from Maron MS et al.[53]**(E) **multiple accessory papillary muscles, 4 in number (arrows); Reproduced with permission from Harrigan C et al.[54]**(F) **7-year-old asymptomatic genotype positive/phenotype negative HCM girl with 3 deep myocardial crypts in the basal (posterior) inferior LV free wall. Ao = aorta; RV = right ventricle; LA = left atrium; LV = left ventricle; VS = ventricular septum

Historically, a number of important limitations of 2-D echocardigraphy have made it difficult to accurately characterize the presence of RV morphologic abnormalities in patients with HCM. However, CMR has demonstrated a number of abnormalities including increased maximal RV wall thickness (ie., ≥ 8 mm) in over one-third of HCM patients and in a substantial proportion of patients RV wall mass is also increased [47,48]. Areas of increased RV wall thickness are most commonly seen near the junction of the insertion of the RV wall into either the anterior or posterior septum (Figure 4a), although involvement of the entire RV does occur [47]. The totalities of these CMR-based observations expand on previous echocardiographic observations by demonstrating that the spectrum of phenotypic expression in HCM also includes morphologic abnormalities of the RV, although the prognostic significance of RV hypertrophy in HCM remains uncertain.

**Figure 5:**
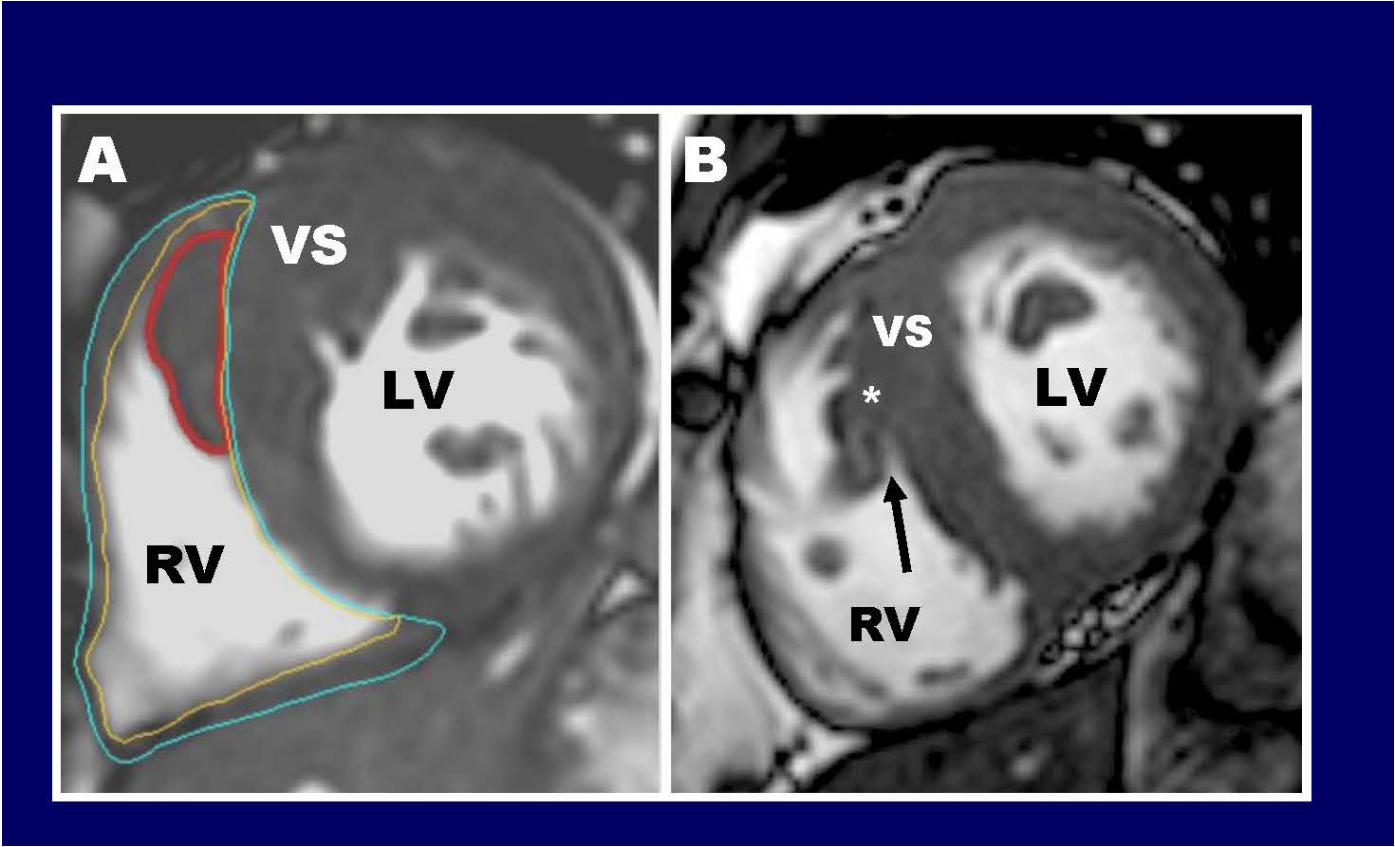
**Right ventricular crista supraventricularis in HCM**. When determining. where to measure the maximal LV wall thickness, it is important to be aware that HCM patients often have prominent and hypertrophied right ventricular muscular structures, the most common of which is the crista supraventricularis. In some HCM patients, the crista supraventricularis is not only significantly hypertrophied (panel **A**, outlined in red) but not uncommonly inserts from its origin in the RV cavity to directly adjacent to the ventricular septum. As a result, this RV muscle structure may be inappropriately included in the measurement of septal thickness---resulting in an overestimation of the maximal LV wall thickness; Reproduced with permission, from Maron MS et al.[47]. In order to avoid including the crista supraventricularis as part of the septum, close inspection of the CMR short-axis cine images can help clarify this issue; (**B) **in a different HCM patient than panel A, the crista supraventicularis muscle is noted to move away from the septum toward the RV cavity with a small area of blood pool noted between the crista and septum (arrow), allowing for a more accurate delineation of the epicardial border of the septum (asterisk).

In addition to RV hypertrophy, CMR can also identify prominent RV muscle structures, such as the crista supraventricularis (Figure 5a). On the basal short-axis images this RV muscle structure is frequently situate adjacent to the ventricular septum and therefore incorrectly included in the measurement of maximal LV wall thickness (potentially resulting in an overestimation of wall thickness measurements; Figure 5b) [47]. However, with close visual inspection of the contiguous stack of short-axis cine CMR images, the crista muscle structure can be seen on certain short-axis slices to separate from the septum in diastole exposing the epicardial border of the ventricular septum allowing one to more easily delineate the epicardial border of ventricular septum (Figure 5b). In addition, HCM patients can develop subpulmonic RV obstruction due to substantial narrowing of the RV outflow tract from excessive hypertrophy of the RV free wall and ventricular septum. In those HCM patients considered for surgical relief of RV outflow tract obstruction, CMR can characterize the precise location and extent of hypertrophy in this region providing the surgeon information to help guide pre-operative surgical management.

#### LV apical aneurysms

HCM patients with thin-walled apical aneurysms associated with mid-ventricular hypertrophy represent an important subgroup of HCM patients who had been under diagnosed prior to the application of CMR to HCM (Figure 4b) [33,49,50]. This is largely due to the fact that small to moderate sized apical aneurysms may not be reliably detected with echocardiography for the same reasons that apical hypertrophy can also be missed [43,44,50,51]. Contrast-enhanced CMR has demonstrated that apical aneurysms are composed predominantly of fibrosis [49,50]. However, LGE often extends from the rim of the aneurysm into the septum and free wall with the junction of these areas representing a nidus for the generation of ventricular tachyarrhythmias [52]. Indeed, these morphologic changes related to the apex are likely what places these HCM patients at increased risk of arrhythmic sudden death and stroke and thus represent a high-risk subgroup. Therefore, the diagnosis of patients with apical aneurysm directly impacts management recommendations for these patients, including consideration for ICD therapy and/or coumadin for stroke prophylaxis [49-51].

#### Mitral Valve

CMR has demonstrated that mitral valve abnormalities represent a primary phenotypic expression of this complex disease, suggesting that pathophysiologic pathways other than those related to the primary sarcomere disease-causing mutation may be important in contributing to aspects of HCM disease expression independent of LV hypertrophy [53]. Mitral valve leaflets are increased in length in many HCM patients, including over one-third of patients with substantially elongated anterior (≥ 30 mm)(Figure 4d) or posterior mitral leaflet lengths (≥ 17 mm) [53]. Leaflet lengths are elongated independent of a number of important HCM disease variables including: age, LV thickness or the presence of outflow tract obstruction. Therefore, these morphologic valvular abnormalities likely represent a primary phenotypic expression of this disease.

Elongated mitral valve leaflets also contribute substantially to the mechanism responsible for subaortic gradients, particularly in those select HCM patients in whom the mitral leaflet length exceeds 2-fold the transverse dimension of the outflow tract at end-systole [53]. Therefore, substantially elongated mitral valve leaflets are an important determinant of LV outflow tract obstruction in some HCM patients, with implications for management strategies in this disease (See surgical myectomy section below).

#### Papillary Muscles

CMR has also expanded our appreciation of other morphologic abnormalities in patients with HCM in addition to those of the LV and RV wall, including the fact that abnormalities in papillary muscle morphology are common in HCM disease expression [54,55]. HCM patients frequently have an increase in the number of papillary muscles, including close to half of patients with 3 or 4 papillary muscles (Figure 4e) [54,55]. Hypertrophy of the papillary muscles is also common, including greater than half of HCM patients demonstrating a 2-fold greater papillary muscle mass compared with than controls. Furthermore, there appears to be a subgroup of HCM patients with normal LV mass (but with localized increase in wall thickness), who nevertheless showed substantially hypertrophied papillary muscles with increased mass (20% of patients) [54]. In such patients, the cardiomyopathic process either disproportional involved papillary muscles (compared to the LV wall), or preferentially affected the papillary muscles. Therefore, similar to the RV, LV papillary muscles appear be part of the cardiomyopathic process in HCM.

### Genotype Positive/Phenotype Negative HCM Patients

The recent penetration of commercial genetic testing has resulted in the increased recognition of family member of HCM patients who carry a disease-causing sarcomere mutation but without LV hypertrophy (genotype positive/phenotype negative [G+/P-]) [17,21,23]. A variety of potential morphologic abnormalities may be present in G+/P- HCM patients and identified by CMR. The most recent of these has been the detection of myocardial crypts (ie., narrow, deep blood-filled invaginations within LV myocardium) localized predominantly to the basal posterior septum and LV free wall (Figure 4f) [56,57], although additional studies are needed to determine precisely how common crypts are in G+P- HCM patients compared to individuals without cardiovascular disease. In addition, elongated mitral valve leaflets [53], and LGE [58-60] may also be markers for gene positive status in HCM family members in the absence of LV hypertrophy. These CMR-based findings represent important clinical and morphologic abnormalities, which expand our current appreciation with respect to disease expression of G+ P- patients and provide evidence that even in the absence of hypertrophy hearts may be morphologically abnormal in this novel and emerging subgroup of HCM patients.

Furthermore, these observations support an expanded role for CMR in earlier diagnosis of relatives within HCM families [23,58]. For example, the identification of one of these morphologic abnormalities by CMR in a relative of an HCM patient in whom genetic testing cannot be performed due to cost or other considerations (or the mutation remains undefined even after testing), should prompt close surveillance with imaging studies to detect development of the phenotype. Also, in patients who can undergo genetic testing, identification of one of these morphologic abnormalities underscores the importance of pursuing genotyping to achieve definitive HCM diagnosis [17,21,23].

### Role for CMR in Differential Diagnosis of LV hypertrophy

#### Metabolic and Infiltrative Cardiomyopathies

Although sarcomeric HCM accounts for the majority of unexplained left ventricular hypertrophy seen in adults, a number of other non-sarcomeric diseases can produce increased wall thickness of the myocardium as part of their phenotypic expression. Infiltrative cardiomyopathies such as cardiac amyloidosis, glyocogen/lysosomal storage diseases including Fabry's, Danons, and AMP kinase are considered to be the most common non-sarcomeric diseases in which cardiac phenotypic expression can mimic that of HCM (ie., "phenocopies") [61]. Fabry's disease is an X-linked storage disease in which mutations in the α-galactosidase A gene lead to cellular accumulation of glycoshpingolipids in multiple organs, including the heart. Cardiac manifestations typically include left ventricular hypertrophy, valvular disease, atrial/ventricular arrhythmias and chest pain due to microvascular ischemia [61]. Although cardiac manifestations can occur early in life, they are generally not detected until the third or fourth decade but remain a major cause of death in patients with Fabry's [61]. Danon disease is another X-linked lysosomal storage disease which can also result in systemic manifestations with associated cardiomyopathy. Clinical suspicion of Danon disease can be raised when young patients present with LV hypertrophy and a 12-lead electrocardiogram with pre-excitation pattern (ie., Wolf-Parkinson-White syndrome) [62]. Rapid clinical deterioration has been observed in patients with Danon disease (often occurring < 25 years of age), leading commonly to heart failure death and even sudden cardiac death [62]. Although these diseases most often have non-cardiac signs and symptoms, in rare instances disease expression can be confined to only the heart [62].

An accurate diagnosis early in clinical presentation is critical, as treatment strategies and prognosis may differ for these diseases compared to HCM [24]. CMR may raise suspicion that a patient does not have HCM, when diagnosis remains uncertain after traditional imaging with two-dimensional echocardiography. For example, the demonstration with cine CMR of nearly identical increased wall thickness measurements in both the septum and LV free wall (ie., "concentric") combined with global subendocardial LGE on contrast-enhanced images is highly specific for cardiac amyloidosis [63]. A similar pattern of concentric wall thickening with LGE confined to the basal inferolateral wall has been frequently reported in Fabry disease [64]. Although CMR findings may suggest that the etiology of LV hypertrophy in an individual patient may be due to a metabolic/infiltrative cardiomyopathy rather than sarcomeric HCM, no pattern of LV wall thickening or LGE is pathogeumonic and therefore confirmatory diagnosis of these diseases ultimately requires the identification of a disease-causing mutation with genetic testing or typical histopathology on cardiac biopsy [17].

#### LV noncompaction

Furthermore, due to its super spatial resolution in imaging the distal LV myocardium, CMR may also help clarify (and even alter) diagnosis by demonstrating the presence of prominent trabeculations (ie., sinusoids) consistent with a diagnosis of LV noncompaction in patients initially diagnosed with apical HCM [65]. In this regard, the LV trabeculations associated with LV noncompaction may appear as apical hypertrophy when imaged with lower spatial resolution two-dimensional echocardiography, potentially resulting in a misdiagnosis of apical HCM in these patients. This also has important implications for management strategies as a diagnosis of LV noncompaction may have additional impact on treatment strategies (ie., coumadin) [66].

#### Hypertensive Cardiomyopathy

Differentiating HCM from wall thickening due to hypertension has historically represented a clinical challenge. Invariably, exposure to long-standing systemic hypertension will result in nearly identical wall thickening in the septum and LV lateral wall (ie., concentric hypertrophy). In addition, hypertensive cardiomyopathy is very rarely associated with resting LV outflow tract obstruction due to typical systolic anterior motion (SAM) with septal contact. Likewise, LV wall thickening in HCM is almost always asymmetric with resting outflow obstruction present in over one-third of patients.

In addition, a subset of HCM patients demonstrate non-contiguous patterns of LV wall thickening [29]. This morphologic pattern consists of hypertrophied segments separated by regions of non-hypertrophied myocardium, creating abrupt changes in wall thickness in adjacent portions of the wall and a "lumpy" hypertrophic pattern (Figure 2f). Such distribution of LV hypertrophy is most consistent with a genetically determined cardiomyopathic process (such as HCM) rather than those forms of hypertrophy secondary to pressure overload (such as in systemic hypertension), and recognition in selected patients could possibly contribute to resolution of the differential diagnosis between HCM and secondary hypertrophy from hypertension.

CMR can also be particularly helpful in detecting changes in serial measurements of LV wall thickness after treatment with antihypertensives, in which regression of hypertrophy would favor a diagnosis of hypertensive cardiomyopathy. In addition, when the distinction between these two disease entities still remains otherwise ambiguous in an individual patient, detection of HCM in family members who were previously undiagnosed or identification of a sarcomere mutation with genetic testing would provide additional evidence strongly favoring a clinical diagnosis of HCM.

#### Athlete's Heart

In addition, it may also be difficult to differentiate HCM from situations in which increased LV wall thickness results as a secondary response to intense athletic training. CMR can have a role in differentiating these conditions by identifying focal, limited hypertrophy not well visualized by echocardiography [67]. CMR is well-suited to accurately compare maximal LV wall thickness measurements before and after a period of systematic deconditioning. Patients in whom wall thickness regresses greater than 2 mm supports a diagnosis of athletes heart, while hypertrophy that remains unchanged suggests HCM [67]. At present, it does not appear that competitive athletes demonstrate LGE [68] and therefore the presence of LGE may also provide additional information to confirm diagnosis of HCM.

### Additional CMR Considerations

#### Hemodynamic Assessment of Outflow Obstruction

Cine CMR can accurately identify the mechanism of subaortic obstruction in HCM with SAM-septal contact in both long-axis and basal short-axis images. Subaortic obstruction will result in high velocity blood flow in the outflow track area which can often be visualized as a systolic signal void jet (ie., black) in the region of SAM-septal contact [69]. In addition, a posteriorly directed signal void in the left atrium can also be seen and represents mitral regurgitation directed through the gap between the anterior and posterior leaflets.

In HCM patients with outflow tract obstruction, phase velocity flow-mapping sequences can be performed in order to determine the peak velocity of blood flow through the outflow tract as well as to quantify the amount of mitral regurgitation. However, only a small number of studies have assessed the accuracy of CMR-derived LV outflow tract velocities compared to continuous-wave Doppler derived pressure gradients [69]. Therefore, it is not certain how well CMR-derived outflow tract velocities correspond to velocities obtained by the standard method of Doppler echocardiography and assessment with CMR can only be made under resting (basal) conditions. This represents a limitation for relying on CMR for gradient assessment as one-third of HCM patients have outflow obstruction only during provocation (ie., exercise). However, a recent study demonstrated that CMR planimetery of the LV outflow tract diameter could differentiate HCM patients with rest (or provokable) outflow tract obtstruction from nonobstructed [69]. Nevertheless, at the current time, clinical management decisions related to outflow obstruction should still likely be based on pressure gradients derived from Doppler echocardiography or hemodynamics obtained during coronary catheterization [41].

#### Surgical septal myectomy

HCM patients with LV outflow obstruction gradient ≥ 50 mmHg at rest or with provocation, who have advanced heart failure refractory to medical therapy, are candidates for invasive septal reduction therapy to relieve obstruction and improve limiting symptoms [3,70]. Surgical myectomy is considered the "gold standard" for the treatment of outflow obstruction [3]. Contemporary surgical strategy requires a thoractomy followed by an aortotmy providing the surgeon direct visualization of the LV outflow tract morphology and the opportunity to resect between 3-12 grams of ventricular septum creating a basal septal trough, which widens the outflow diameter providing virtually complete elimination of outflow gradients in the vast majority of patients (and a substantially reduction in mitral regurgitation), resulting in a significant and long-lasting improvement in heart failure symptoms [71].

CMR has a role in pre-operative surgical myectomy planning in those HCM patients who are considered candidates for the operation by characterizing a number of important morphologic abnormalities related to outflow tract anatomy as well as the mitral valve and submitral apparatus [41,53-55]. For example, the identification of the pattern and distribution of wall thickening in the basal septum at the point of SAM-septal contact (particularly when these structures are not well visualized with echocardiography) can provide the surgeon an accurate estimate to the depth of the extended surgical resection of septal muscle necessary to achieve optimal relief of outflow obstruction [41].

CMR can identify additional morphologic abnormalities of the mitral valve and papillary muscles, which are important contributors to the mechanism responsible for subaortic gradients. Specifically, in those patients with substantially elongated mitral valve leaflets (Figure 4d) the mitral-septal contact point (and site of subaortic obstruction) can be displaced distal to its usual position. As a result, surgical strategy in this case may be altered to address this situation, by promoting extended muscular resection as well as the possibility of a combined approach of septal myectomy and mitral valve repair, with leaflet extension or plication, in order eliminate SAM of the mitral valve and absolute reduction of outflow gradients [53,72,73].

In addition, a number of other structural abnormalities of the submitral apparatus notable for proper surgical myectomy planning can be routinely identified by CMR. Apically displaced accessory anterolateral (Figure 4e) or a double bifid papillary muscle act to tether the plane of the mitral valve toward the ventricular septum facilitating the drag phenomenon of systolic anterior motion (SAM) and as a result are associated with a significantly higher likelihood of having outflow tract obstruction [54,55]. As a result, the surgeon, as part of an extended septal myectomy, often resects these papillary muscles in order to ensure a pristine hemodynamic result. In addition, anomalous insertion of the papillary muscle directly into the anterior leaflet of the mitral valve in the absence of chordae tendinae will result in mid-systolic apposition of the papillary muscle and ventricular septum resulting in mid-cavitary outflow obstruction (Figure 4c) and therefore often require distal resection of the basal septum as well as surgical revision of the abnormal papillary muscle [74].

#### Alcohol septal ablation

Alcohol septal ablation (ASA) is an alternative invasive septal reduction therapy in which 1-2 cc of alcohol are injected into an anatomically appropriate septal perforator artery supplying the basal septum (at the point of SAM-septal contact) creating a myocardial infarction resulting (ultimately) in septal thinning, widening of the LV outflow area and reduction of obstruction [75]. For HCM patients under consideration for ASA, there are currently no established CMR-based markers which identify HCM patients more likely to have an optimal hemodynamic result with ASA nor in identifying patients who are at risk for procedural complications such as complete heart block requiring permanent pacemaker implantation. CMR can precisely quantify the amount of tissue necrosis (average of 10% of LV mass) induced by ASA as well as identifying the relationship between the location of scarring and outflow tract morphology as well as accurately assessing regression of LV mass following the procedure [76,77].

### LVH and Risk Stratification

#### LV hypertrophy

Noninvasive imaging of LV wall thickness has proven to have a role in risk stratification with massive LV hypertrophy of ≥ 30 mm demonstrated by 2-D echocardiography anywhere in the LV chamber identifies those HCM patients at highest risk and potentially deserving of ICD therapy for primary prevention of sudden death [26,41,78]. Indeed, the presence of massive hypertrophy alone, in the absence of any additional risk markers, may be enough to recommend ICD therapy for primary prevention of sudden death [26]. Therefore, accurate assessment of maximal wall thickness is an essential part of the initial evaluation of all HCM patients. Previous observations have demonstrated that CMR can identify massive LV wall thickening (≥ 30 mm), confined to the anterolateral free wall, which was substantially underestimated in magnitude by 2-dimensional echocardiography (Figure 3c and 3d) [42].

#### LV Mass

Due to the variable distribution of LV hypertrophy in regions of the LV chamber remote from maximal wall thickness, CMR-derived LV mass provides the most accurate assessment of the overall extent of LV hypertrophy in this disease. As a result, LV mass may represent a marker for adverse risk and would therefore seem to hold promise for aiding in risk stratification [46]. However, long-term prospective CMR studies are needed before establishing the precise relationship between LV mass and outcome in this disease.

### Significance and Clinical Implications of LGE

**Figure 6:**
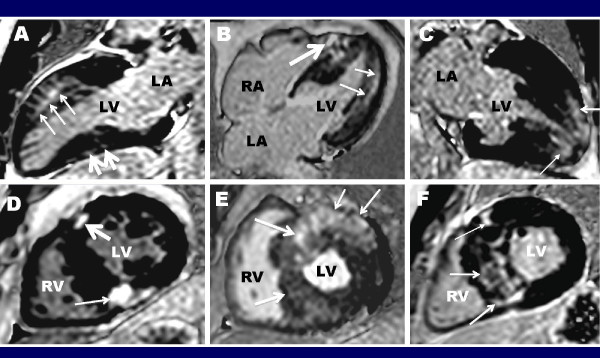
**Contrast-enhanced CMR images in 6 different HCM patients demonstrating the diverse pattern and extent of late gadolinium enhancement in this disease**. **(A) **extensive transmural LGE in the anterior wall (small arrows) with smaller focal area in the inferior wall (small arrows); **(B) **mid-myocardial LGE in the lateral wall (small arrows) and diffuse LGE in the ventricular septum which extends into the RV wall (large arrows) in a 26 year-old man with "end-stage" phase of HCM with an ejection fraction of 40%; **(C) **LGE confined to the LV apex (arrows); **(D) **LGE localized to the insertion area of the RV wall into the anterior (large arrow) and posterior ventricular septum (small arrow); **(E) **transmural LGE involving the majority of the ventricular septum (large arrow) and lateral wall (small arrow). **(F) **Basal short-axis image with transmural LGE located predominantly in the ventricular septum (arrows). RA = right atrium; RV = right ventricle; LA = left atrium; LV = left ventricle

Recently, considerable interest has emerged in using non-invasive *in vivo *detection of myocardial fibrosis as a prognostic marker in HCM [26,28,30,32]. Following the intravenous injection of gadolinium, contrast-enhanced CMR techniques can be applied to patients with HCM to detect areas of LGE, which can be planimetered and the amount quantified and expressed as a % of the total LV mass. Depending on the selection of patients studied and which quantification technique used, between 50-80% of HCM patients have been reported to demonstrate LGE and when present occupying on average 10% of the overall LV myocardial volume (Figure 6) [36-39].

#### Pathophysiology of LGE

The precise pathophysiologic mechanism responsible for LGE in HCM currently remains uncertain. Nevertheless, observations derived from contemporary imaging and histologic studies provide support for the principle that LGE may derive from a pathophysiologic cascade in which repetitive bouts of microvascular ischemia result from structurally abnormal intramural coronary arteries (with impaired vasodilatory capacity) responsible, over a period of time, for myocardial ischemia-mediated myocyte death, ultimately triggering repair in the form of replacement fibrosis [79].

Indeed, there are a number of avenues of support for the principle that LGE probably constitutes (or largely represents) areas of myocardial replacement fibrosis. Studies in HCM patients with stress-CMR and PET have demonstrated myocardial blood flow to be substantially reduced in LV segments occupied by LGE and severely blunted in areas situated adjacent to LGE [80,81]. In ventricular septal tissue removed from HCM patients at the time of surgical myectomy, there is a strong association between the presence (and extent) of abnormal intramural coronary arteries (by histologic examination) and LGE (as determined from preoperative contrast-enhanced CMR studies) [82]. As well, blunted myocardial blood flow has also been shown to be an independent predictor of the end-stage phase of HCM (EF < 50%),[83] a phenotype associated with substantially amounts of myocardial fibrosis[84]. Indeed, the only reports correlating histologic evidence to CMR findings of LGE in HCM are a small number of case reports describing end-stage HCM patients in which LGE correlates to areas of fibrosis [85,86].

#### Pattern and distribution of LGE

Virtually any pattern, distribution and location of LGE can be observed in HCM, although LGE never corresponds to a coronary vascular distribution (Figure 6)[37,87,88]. LGE is most commonly located in both ventricular septum and free wall (over 30% of patients), but less commonly can be confined to the free wall, septum, apex, and the areas of right ventricular insertion into ventricular septum [37]. In addition, LGE can also occur in other structures outside of the LV, including RV wall [47,48] and isolated to the papillary muscles [54], suggesting that a similar process of myocardial fibrosis which occurs in the LV can also occur (although much less frequently) in other areas of the heart.

Transmural extent of LGE in not uncommon, occurring in one-half of HCM patients [37]. A significant but modest relationship is present between hypertrophy and LGE. Patients with LGE have greater maximal LV wall thickness and LV mass index than patients without LGE [37,87,88]. On an individual patient basis, a relationship is also present between segmental LV wall thickness and LGE [37,88].

#### Adverse LV remodeling

One of the strongest and most consistent observations derived from a number of contemporary cross-sectional studies is the inverse relationship between ejection fraction and LGE in HCM. LGE extent is greatest in HCM patients with EF < 50%. (ie., end-stage phase) while those with hyperdynamic LV systolic function have comparatively minimal LGE [37,87,88]. However, HCM patients with low-normal EF (50-65%) show amounts of LGE that overlap those in the end-stage phase, and also demonstrate LV cavity dimensions that are more similar to the end-stage than patients with intact systolic function [89]. These observations would suggest some HCM patients with low-normal LV function are at risk of progressive fibrosis transitioning to a phase of more advanced LV remodeling associated with systolic dysfunction. However, due to the relatively recent introduction of CMR into cardiology practice, longitudinal follow-up studies characterizing the development and natural history of LGE in HCM are not yet available. Consequently, it is not possible at this time to determine if a threshold amount of LGE exists which identifies HCM patients at future risk of progressive remodelling. Nevertheless, these observations have potentially important clinical implications as HCM patients with systolic dysfunction represent a high risk subgroup at increased risk of sudden death and advanced heart failure symptoms prompting consideration for ICD therapy and alternative medical therapy e.g., ACE-inhibitors or aldosterone inhibitors to improve adverse LV remodelling [84]. Therefore, HCM patients identified with low-normal LV ejection fraction should undergo close clinical follow-up with serial imaging for prospective detection of changes in symptoms and LV structure.

#### Heart failure symptoms with preserved EF

At present, the precise impact of LGE on the development of heart failure symptoms is unresolved. A few studies have reported a weak but significant relationship between LGE and progressive heart failure symptoms/death in HCM (and the risk proportional to the extent of LGE) [37,38]. However, in one such study, the clinical relevance of this relationship was driven predominantly by unplanned hospitalizations, a "soft" clinical end-point in HCM [38]. In addition, at this time it is not established that LGE provides additional information with respect to future risk of heart failure symptoms beyond the currently established predictors of symptom progression in this disease such as LV outflow tract obstruction [90].

#### Sudden Death

**Figure 7:**
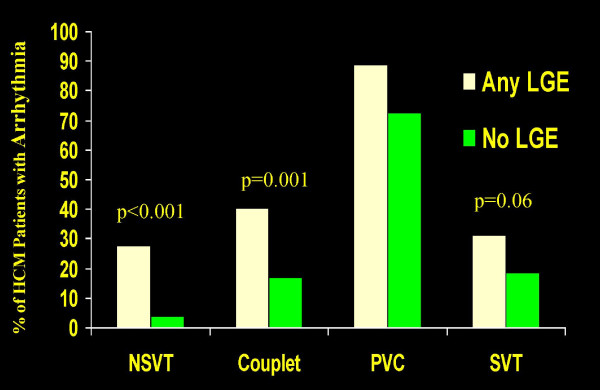
**Prevalence of arrhythmia on 24-hour Holter ECG with respect to presence of late gadolinium enhancement in patients with HCM. Adapted with permission, from Adabag et al.**[93]. ECG = electrocardiogram; HCM = hypertrophic cardiomyopathy; LGE = late gadolinium enhancement; NSVT = nonsustained ventricular tachycardia; PVC = premature ventricular contraction; SVT = supraventricular tachychardia

Despite considerable advances, risk stratification in HCM remains incomplete as some at-risk patients are not identified by the conventional risk factors suggesting a need to identify novel markers of susceptibility to sudden death risk [12,26,91,92]. Therefore, in this regard, there is substantial interest in exploring other modalities such as contrast-enhanced CMR. Indeed, based on recent cross-sectional studies, a strong association has been demonstrated between LGE and ventricular tachyarrhythmias on ambulatory 24-hour Holter ECG (Figure 7) [39,93-97]. Up to a 7-fold increased risk for potential lethal ventricular tachyarrythmias has been demonstrated among HCM patients with LGE compared to those without LGE [93]. These observations would suggest that LGE may represent an unstable arrythmogenic substrate responsible for ventricular tachyarrythmias in HCM.

Since nonsustained ventricular tachycardia is an independent risk factor for sudden death in this disease [98], the relationship between ambulatory ventricular tachyarrhythmia and LGE support the possibility that contrast-enhanced CMR could represent a novel risk marker and thereby improve current risk stratification strategies by identifying HCM patients at increased risk of sudden death. In this regard, 4 prospective (short-term) outcome studies with relatively small number of HCM patients have been published that have demonstrated conflicting results regarding the relation between LGE and sudden death and/or appropriate therapy for ventricular tachycardia/fibrillation (table 1) [36-39]. When data from these studies were combined, LGE was more common in patients who experienced sudden death or an appropriate ICD discharge than those who did not resulting in a significant but weak relationship between the presence of LGE and risk for sudden death (table 1).

**Table 1:** Comparison of 4 short-term follow-up contrast-enhanced CMR studies in HCM

Study	No. Patients	LGE Quantification Technique	Total SD Events	LGE Positive with SD	LGE Negative with SD	p-value
**Bruder et al 2010**	**220**	**≥ 2 SD** **Threshold**	**11**	**10**	**1**	**0.1**

**O'Hanlon et al 2010**	**217**	**FWHM**	**4**	**3**	**1**	**1.0**

**Rubinshtein et al 2010**	**424**	**Visual**	**8**	**8**	**0**	**0.002**

**Maron et al 2008**	**202**	**≥ 6 SD Threshold**	**7**	**4**	**3**	**0.05**

**Overall**	**1063**	**--------------**	**30**	**25**	**5**	**0.04**

Abbreviations: FWHM = full width half maximum; LGE = late gadolinium enhancement; No. = number; SD = standard deviation

**Figure 8:**
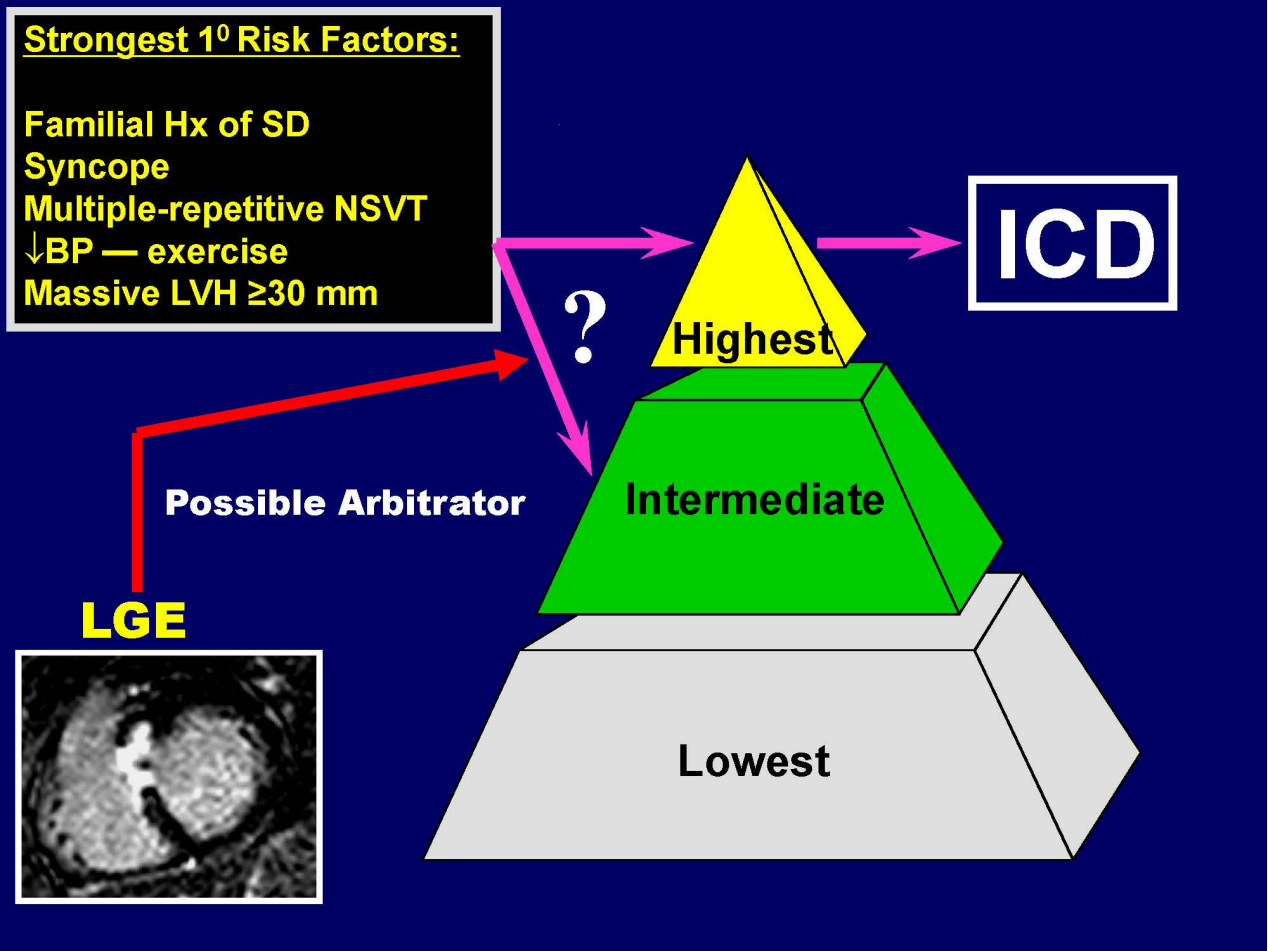
**Role of CMR in Sudden Death Risk Stratification**. Results of contrast-enhanced CMR with late gadolinium enhancement could be used as a potential arbitrator to arrive at a decision regarding ICD therapy for primary prevention of sudden death in HCM patients in whom risk still remains ambiguous after assessment with current conventional risk factors.

Therefore, available data do not strongly support LGE as a primary independent risk factor for sudden death in HCM [26]. As a result, recommendations for primary prevention ICDs should not be based solely on the presence of LGE in individual patients. Nevertheless, given the strong association of LGE and ambulatory ventricular arrhythmias, it is not unreasonable to give some weight to this finding as an arbitrator in reaching recommendations for prophylactic ICDs in selected patients in whom other evidence of risk is ambiguous (Figure 8) [93].

The clinical significance of LGE is currently one of the most important and debated clinical questions in HCM [28,99]. Resolving this issue will require demonstrating that LGE is a clinically relevant marker of sudden death in large, prospectively selected cohorts with substantial number of patients followed over periods of time sufficient to ultimately provide the statistical robustness to establish the independent contribution of LGE to sudden death (in what is essentially a low-risk disease overall). In addition, it is imperative that future studies comparing LGE to outcome only employ established well-defined end-points clinically relevant to HCM, as well as compare the predictive value of LGE to the 5 established noninvasive risk markers currently used to identify patients at-risk.

Another point in establishing clinical relevance for contrast-enhanced CMR is the relationship of presence vs. extent of LGE with respect to outcome. The majority of follow-up contrast-enhanced CMR studies have reported only an association between the presence of LGE and sudden death [36,37,39]. However, the reported prevalence of LGE is over 50% (up to 80% in one study)[97]. Therefore, even if some relationship can be derived between the presence of LGE and sudden death, LGE alone would not qualify as a practical risk marker as simply too many HCM patients would be identified for primary prevention ICDs. Therefore, for contrast-enhanced CMR to be a clinically useful tool for management decisions, it will likely be necessary to ultimately demonstrate that risk of adverse outcome is related to *extent *of LGE or when LGE is combined with the other currently established prognostic markers into a composite risk score for sudden death.

### LGE Disclaimers in HCM

#### Does LGE always represent myocardial fibrosis in HCM?

Over the last several years, it has become increasingly common in the HCM literature and clinical arena to equate areas of LGE directly to myocardial fibrosis [28,82,85-88,93,95,100]. This assumption is largely based on extrapolation from CMR-based animal models of myocardial fibrosis in which areas of fibrosis from the core infarct correlate to areas of LGE[101], as well as the association in HCM patients between areas of abnormal myocardial blood flow and hyperenhancement [79,81,102]. Yet in HCM, a number of obstacles continue to make it difficult to understand the precise histologic basis of LGE in this disease, including lack of a suitable HCM animal model and difficulty in obtaining post-mortem and explanted hearts for histopathologic examination close to the time of a prior clinical CMR examination. Indeed, the only correlative studies which directly relate CMR and histopathologic findings are derived from explanted end-stage HCM hearts [85,86], making it difficult to extrapolate those findings to more typical HCM patients with normal preserved EF. However, these studies do show extensive amounts of fibrosis responsible for the adverse LV remodelling in this subgroup of HCM patients [85,86].

However, a number of CMR observations in HCM would suggest that, in fact, not all LGE may represent myocardial fibrosis in this disease. First, LGE in HCM is more commonly found in those LV segments which are the thickest with normal regional systolic function [37,87-89]. This observation is in direct opposition to that in patients with ischemic heart disease, in which LV segments with LGE are often thinned with associated wall motion abnormalities [103]. Second, extensive amounts of LGE can be present in asymptomatic HCM patients who have achieved advanced age (> 60 years) with normal systolic function and without adverse disease consequences such as potentially lethal arrhythmias, heart failure symptoms or adverse LV remodelling [37]. Furthermore, among end-stage HCM patients the amount of LGE often exceeds that observed in any other cardiovascular disease, including patients who have suffered large ST-segment elevation myocardial infarction suggesting that even within this subgroup of patients not all LGE maybe myocardial scar [84,89].

Therefore, based upon the totality of these observations it would be reasonable to consider that some LGE in HCM may represent histopathology which is not a result of myocardial scarring from replacement fibrosis. For instance, gadolinium may deposit within the diffuse interstitial collagen (matrix) compartment between normally aligned myofibrils or areas of perivascular fibrosis. Matrix collagen constitutes a greatly expanded extracellular tissue volume partially responsible for increased LV wall thickness, represents a primary morphologic feature of HCM, and provides an opportunity for gadolinium accumulation that may not reflect true replacement fibrosis. In addition, disorganized myocyte architecture will result in expanded areas of myocardial matrix where gadolinium could aggregate. Consistent with this point is the fact that one of the most common locations of focal LGE in HCM is at the insertion of RV wall into anterior or posterior ventricular septum [37,39,87,88]. Kuribayashi et al.[104] has shown in post-mortem HCM hearts that this particular area of the LV chamber is characterized histologically by an expanded extracellular space created by the arrangement of intersecting myocardial fibers (at the juncture points of both the LV and RV) and therefore accumulation of gadolinium in this area is unlikely to represent replacement fibrosis.

With current CMR technology it is not possible to reliably distinguish interstitial (matrix) from replacement fibrosis, or an expanded matrix compartment created by myocyte disarray [100]. This may be an important limitation to contrast-CMR, as these forms of histopathology in HCM may portend different clinical consequences, particularly regarding susceptibility to potentially life-threatening arrhythmias.

Nevertheless, there has been interest in using CMR to differentiate myocardial substrates of different histologic composition. This principle has been addressed predominantly in patients with atherosclerotic coronary artery disease and prior myocardial infarction, in which areas of intermediate LGE signal intensity (ie., "grey-zone") correlate histologically to regions of 'tissue heterogeneity' (mixture of isolated myocytes and fibrosis), while regions of higher signal intensity LGE correlate with core infarct zones comprised of only replacement scar[105]. Identification of areas of tissue heterogeneity are of potential importance, as these regions may represent a more arrthymogenic substrate than the core infarct [106].

In HCM, areas of lesser signal intensity LGE are also common but have largely been ignored in prior analysis of hyperenhancement in this disease and therefore their potential for generating life-threatening ventricular arrhythmias largely ignored [37-39,89,93]. However, a recent investigation in HCM, applying a similar imaging strategy to that used in ischemic cardiomyopathy to identify the "grey zone" border surrounding core infarct, demonstrated that magnitude of intermediate LGE signal intensity (≥ 4 but < 6 SD above the mean SI of nulled myocardium) was a more reliable LGE discriminator for identifying HCM patients with ambulatory complex arrhythmias such as NSVT than high signal intensity LGE (≥ 6 SD) [107]. These data underscore the potential value in assessing regions of intermediate signal intensity LGE, which may identify another abnormal myocardial substrate more prone to ventricular tachyarrhythmias. In order to resolve this issue, further longitudinal studies are necessary to assess whether the character of LGE (intermediate vs. higher signal intensity) is a predictor of clinical outcome in HCM patients with preserved systolic function.

#### Quantification of LGE in HCM

**Figure 9:**
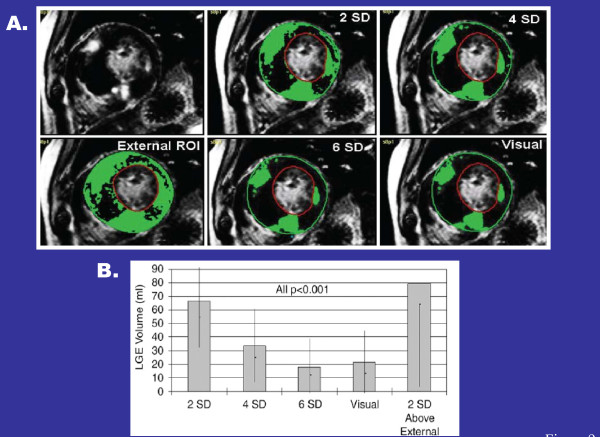
**Quantification of LGE**. (**A) **Identical LV short-axis contrast-enhanced cardiovascular MR images show LGE depicted with gray-scale thresholding techniques at 2, 4, and 6 SDs above mean signal intensity of normal remote myocardium, with visual assessment, and with 2 SDs above mean of external region of interest *(ROI)*. LV endocardium and epicardium are outlined in red and green, respectively. Solid black areas in LV myocardium represent areas of delayed enhancement at corresponding semiautomated threshold; **(B) **Graph illustrates volumes of delayed enhancement (horizontal axis values) assessed by using various gray-scale thresholding techniques (2, 4, and 6 SDs above mean), visual assessment, and an external region of interest. Reproduced with permission, from Harrigan C et al.[113]

Over the last several years, numerous techniques have been proposed to assess extent of LGE in HCM reflecting the uncertainty which persists regarding the most appropriate strategy for assessing hyperenhancement in this disease [108-112]. The most widely used techniques have been a variety of semi-automated algorithms which identify high signal intensity LGE pixels after applying a grayscale threshold a number of standard deviations (SD) above the mean signal intensity within a remote region of interest (ROI) containing normal "nulled" myocardium (i.e. 2, 4, 5 or 6 [SD])(Figure 9a) or peak intensity of scarred myocardium (i.e. full width at half maximum [FWHM]). In addition, the Raleigh curve method is a novel quantitative technique which addresses limitations inherent in defining a ROI (ie., the assumption that within an ROI the signal intensity pixels always conform to a Gaussian distribution) by placing an ROI in the background of the image to generate a more ideal myocardial signal intensity distribution curve [108].

These thresholding algorithms has been most extensively studied in patients with ischemic cardiomyopathy, where grayscale techniques using 2 SD above the mean of normal myocardial SI or FWHM have been shown to correlate well with the spatial extent of the infarct [101,113]. In addition, because the infarct scar is surrounded by an otherwise structurally normal myocardium there are no significant differences in the amount of LGE if a 2SD threshold is applied compared to using other grayscale thresholds.

However, HCM represents a distinctly different disease entity in which abnormal myocardial substrate (interstitial-matrix, replacement fibrosis and areas of myocyte disarray) involve large proportions of LV myocardium [106,114-116]. Therefore, directly extrapolating fundamental principles of LGE analysis from patients following CAD-related myocardial infarction to those with HCM may incorporate flawed assumptions [111]. For example, in an individual HCM patient there may be significant differences in amount of LGE identified depending on which greyscale threshold technique is chosen, with a 2SD threshold resulting in a 2-fold greater amount of hyperenhancement compared to a 6SD threshold (Figure 9B) [108,111,112]. However, 6 SD and FWHM have been found to most closely approximate the extent of fibrosis compared with visual assessment (or the Raleigh curve method) and to be the most reproducible method for quantification of LGE in HCM compared to other grayscale thresholds (Figure 9b) [108,111,112]. Therefore, in the absence of data comparing LGE images to that of *ex vivo *histopathology in an HCM patient with preserved EF (or appropriate animal models), it seems most reasonable at this time to promote the use of either of these two methods for quantifying LGE in HCM.

Furthermore, there are a number of technical issues to consider as potential limitations with respect to LGE images. The quality of contrast-enhanced images can be affected by the selection of poor inversion times and motion artifact, making it difficult to reliably determine in such cases whether LGE represents abnormal histopathology versus "background noise" [117]. In addition, a number of different types of sequences are now being used to acquire LGE images, including 3-D segmented inversion-recovery, phase-sensitive inversion-recovery and equilibrium contrast CMR [117-119]. There are currently no data available which compare the extent of LGE identified using these different techniques, and there is variability among individual operators with respect to optimizing respective image parameters. The limitations with respect to quantification of LGE in HCM is underscored by the fact that the 4 short-term follow-up contrast-enhanced CMR studies in this disease have each applied a different quantification method to determine the presence and/or extent of LGE, which provides an additional explanation for why the results of these outcome studies are so dissimilar [36-39].

At present, the totality of these data suggests a note of caution is appropriate in interpreting the significance of LGE from a histologic perspective in patients with HCM. It is reasonable to conclude that in end-stage HCM patients the majority of LGE represents myocardial replacement fibrosis responsible for the adverse LV remodeling evident. It remains less clear to what extent LGE equates to scar formation among the more typical HCM patients with preserved EF, who comprise the vast majority of patients with this disease. Nevertheless, even if all hyperenhancement does not equate with fibrosis, LGE may still prove to be an important clinical marker for prognosis in this disease. In addition, at this early juncture, there still remains a great need for standardization of LGE quantification methods and sequence acquisition, given that the substantial amount of variability evident among these techniques represents a potential limitation in applying CMR to routine clinical decision-making.

## Conclusions

Over the last decade, the unique imaging strengths of CMR have led to improved diagnostic capabilities and expanded our understanding and appreciation of the diverse phenotypic expression of this complex genetic heart disease. In addition, CMR has an emerging role in the assessment of risk in patients with HCM, as substantially increased LV mass and late gadolinium enhancement have been associated with increased likelihood of future adverse cardiovascular events. Taken together, these observations underscore an important and growing role for CMR in the contemporary evaluation of HCM patients and support the need for future longitudinal studies to clarify whether CMR-derived variables will be independent predictors of sudden death and disease progression.

## List of Abbreviations

CMR: cardiovascular magnetic resonance; EF: ejection fraction; HCM: hypertrophic cardiomyopathy; ICD: implantable cardioverter defibrillator; LGE: late gadolinium enhancement; LV: left ventricle; RV: right ventricle; SAM: systolic anterior motion

## Competing interests

Consultant PGx Health

## Authors' contributions

I have read and approved the manuscript

## Authors Information

Dr. Martin Maron is an assistant professor of medicine and Director of the Hypertrophic Cardiomyopathy Center and Co-Director of Advanced Cardiac Imaging at Tufts Medical Center. His research interests involve the role of cardiovascular magnetic resonance (CMR) in the evaluation and management of patients with HCM as well as investigating novel drug therapy to modify the HCM phenotype. Dr. Maron also collaborates with members of the Molecular Cardiology Research Institute (MCRI) at Tufts on several projects aimed at better characterizing the molecular pathways which contribute to disease expression in HCM. Several of these on-going projects include evaluating the relationship of novel polymorphisms with regard to the expression of hypertrophy and characterizing signaling pathways which promote the upregulation of cardiac fibroblasts and their promotion of adverse remodeling in HCM.
